# Reach-to-Grasp: A Multisensory Experience

**DOI:** 10.3389/fpsyg.2021.614471

**Published:** 2021-02-09

**Authors:** Sonia Betti, Umberto Castiello, Chiara Begliomini

**Affiliations:** Department of General Psychology, University of Padova, Padova, Italy

**Keywords:** multisensory integration, kinematics, grasping, sensory perception, reach-to-grasp

## Abstract

The reach-to-grasp movement is ordinarily performed in everyday living activities and it represents a key behavior that allows humans to interact with their environment. Remarkably, it serves as an experimental test case for probing the multisensory architecture of goal-oriented actions. This review focuses on experimental evidence that enhances or modifies how we might conceptualize the “multisensory” substrates of prehension. We will review evidence suggesting that how reach-to-grasp movements are planned and executed is influenced by information coming from different sensory modalities such as vision, proprioception, audition, taste, and olfaction. The review closes with some considerations about the predominant role of the multisensory constituents in shaping prehensile behavior and how this might be important for future research developments, especially in the rehabilitative domain.

## Introduction

The notion that senses are better conceptualized as interrelated modalities rather than independent channels is supported by several studies, providing evidence for common neural and psychological mechanisms for the processing of multisensory information (e.g., Graziano and Gross, 1993; Driver and Spence, 1998; Spence et al., 2000; Doyle and Walker, 2002). The creation of a unitary percept of objects is one of the classic roles attributed to multisensory integration (Newell, 2004). Indeed, we are able to recognize a mug not only by looking at it but also, for example, by touching it when it is out of view. Similarly, we can recognize a robin redbreast relying on visual information about its size, shape, and colors, but also by hearing its song.

In humans, most of the research conducted on crossmodal integration has typically focused on perceptual integration, studying this phenomenon by using arbitrary responses (e.g., reaction times, saccadic eye movements). Less clear are the effects of multisensory coding during more natural tasks, such as upper limb tasks, where actions are performed in three-dimensional space. Indeed, different sensory modalities are used in concert not only to perceive objects but also to represent actions (Fogassi and Gallese, 2004). In this respect, a fundamental role of multisensory integration is to help in the planning and execution of actions. In fact, most of the actions we perform daily rely on sensory information and, to act appropriately, we often have to process information coming from more than one sensory modality in parallel. The act of kicking a ball, for example, requires the integration of visual, proprioceptive, and tactile modalities. Writing is another example of an action that, to be accomplished accurately, requires the integration of visual, proprioceptive, and tactile information. In addition, recognizing and understanding what other individuals are doing depends on multimodal information (Fogassi and Gallese, 2004). As an example, by hearing the sound made by the flowing water into a glass, we are able to reasonably recognize the act of pouring even without seeing the acting individual. Thus, information arriving through different and multiple sensory modalities can greatly facilitate the retrieval of the representation of a given object, acting individual, or a given action in our brain.

To date, crossmodal links between vision, audition, sense of touch, and proprioception have been extensively documented for grasping an object with hands (Johansson and Westling, 1984; Klatzky et al., 1987, 2000; Klatzky and Lederman, 1988; Goodwin et al., 1998; Jenmalm et al., 2000; van Beers et al., 2002; Patchay et al., 2003, 2006; Sober and Sabes, 2003; Aziz-Zadeh et al., 2004; Gazzola et al., 2006; Zahariev and MacKenzie, 2007; Etzel et al., 2008; Castiello et al., 2010). Here we present a series of studies that demonstrate crossmodal links between vision and other modalities (audition, olfaction and proprioception) during grasping. Prehensile actions are one of the most frequent behavior we perform and represent a remarkable experimental test case to probe the multisensory nature of our behavior. Specifically, in virtue of the well-characterized kinematics profile of reach-to-grasp movements (Castiello and Ansuini, 2009; Jeannerod, 2009) and of the detailed and multifaced information that it can provide, we mainly focus on the contributions coming from the study of grasp kinematics to reveal its multisensory nature. The results indicate a strong multisensory effect on the posture assumed by the hand during a visually guided reach-to-grasp movement, which is also evident at the level of action planning.

## The Role of Tactile Information

How tactile information influences grasping kinematics has been first explored by presenting targets of different dimensions either in visual or haptic modality (Chieffi and Gentilucci, 1993), and asking participants to judge their size and position. Results show that the sensory modality did not affect size estimation for the large target, whereas small stimuli tended to be underestimated when judged relying on tactile information. Adopting a similar approach, Camponogara and Volcic (2019a,b, 2020) recently showed how the role of the sensory modality changes over time when the right hand grasps an object which is perceived through vision, or haptically, with the left hand. When only haptic information was available, wider grip aperture and earlier initiation characterized hand preshaping, whereas the final phase of the action was slower. Conversely, vision appeared to be more relevant for the final phases of the movement, where the hand approaches the object and on-line visual feedback becomes more crucial. Visuo-haptic information made the action more efficient and precise, with the grip aperture becoming less variable and the movement execution faster.

**Figure 1:**
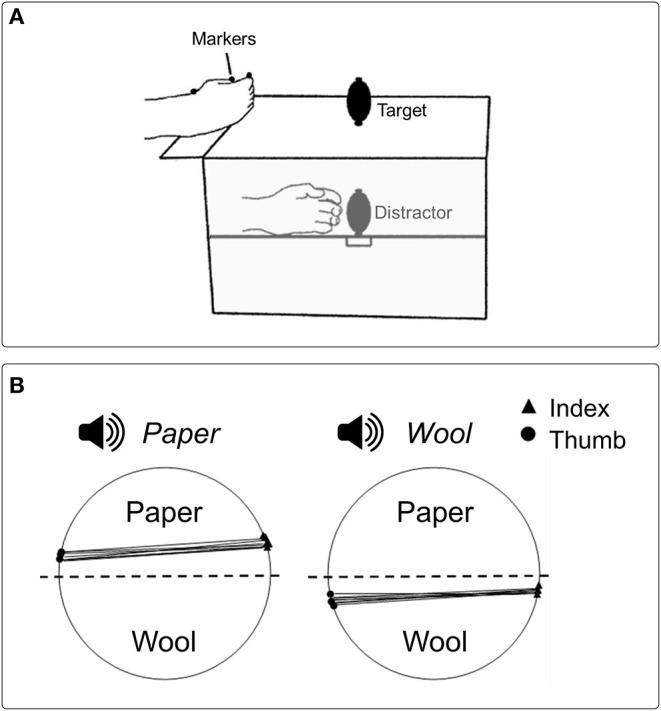
**(A)** Schematic representation of the experimental setup used in Patchay et al. (2003) to study how haptic and proprioceptive inputs coming from an unseen distractor grasped by a non-reaching hand influenced reach-to-grasp actions toward a visual target performed by the other hand. Both the target and the distractor occupied spatially coincident locations, and the distractor could have a smaller or greater size relative to the target. **(B)** Graphical representation of contact points for the index finger and thumb from a representative participant in Castiello's et al. (2010) study, where the influence of contact sound on grasping actions was investigated. A sound congruent with the material covering one of the two parts of a visual target made participants more likely to grasp the object from the surfaced covered by the same material (e.g., paper, wool).

The effect of tactile information on grasping kinematics has been documented also in terms of competing information: Gentilucci et al. (1998) asked participants to reach and grasp visually presented objects presented in different sizes with one hand, while holding another unseen object (i.e., distractor) of different sizes (smaller or greater than the target) in the other hand. The main finding was that the size of the distractor did affect the kinematics of the grasping executed with the other hand: in particular, maximal hand aperture decreased and increased when the distractor was smaller and larger than the visual target-object, respectively. However, the effect of tactile information was observed only when the visual target-object was small and manipulation was performed using the right hand. This rendered unclear what caused the effect. These results have been confirmed and extended with a similar paradigm (Patchay et al., 2003, 2006), showing that maximum hand aperture for the visual target was proportional to the dimension of the distractor, which was manipulated proprioceptively with the other hand (Figure 1A). Analogous patterns were observed when the distractor was manipulated with either the left or the right hand. Noticeably, the effect of tactile information occurred only when the distractor was actively grasped; the effect was absent when the non-reaching hand received passive tactile and proprioceptive stimulation.

Overall these findings show that exploring large or small object activates the movement parameterization which corresponds to the size of that object, i.e., big and small hand aperture, respectively, indicating that the selection of the appropriate “grasp” motor plan for interacting with an object can be based on tactile cues. Therefore, the mechanism underlying the guidance of actions is not only sensitive to the information conveyed via vision but also via the sense of touch. Subsequent studies suggested that the contribution of tactile information better emerges when the inputs coming from the sensory channels disagree: while in conditions of congruency between perceptual inputs (e.g., vision and touch) the benefit of adding tactile information over vision only is almost negligible, in case of a mismatch between sensory inputs a larger variability in performance is observed (Pettypiece et al., 2010).

However, the interaction between vision and touch extends beyond grasp kinematics: for example, studies on haptic “memory” demonstrate that the weight of a previously manipulated target affects forces employed to grasp subsequent objects (Johansson and Westling, 1988; van Polanen and Davare, 2015), and changes the haptically perceived object weight independently of its visual appearance (Maiello et al., 2018). Similarly, presenting asynchronous visual and haptic feedback during object lifting can alter both force scaling and haptic weight perception (van Polanen et al., 2019).

## The Role of Auditory Information

When using hands to manipulate objects and to interact with surfaces we generate contact sounds, providing important information concerned with the interaction between the moving effectors and the acted upon object. In particular, the contact sound signals both functional consequences and completion of manipulative actions. For instance, the “crash” sound associated with our hands breaking a walnut can be considered as a contact sound. Upon hearing the “crash,” we become aware of having broken the shell, thus, we stop the walnut handling, and we bring the husk to our mouth.

Zahariev and MacKenzie (2007) have focused on the role played by contact sounds in hand grasping by asking participants to perform reach-to-grasp actions toward a visual target (i.e., a wooden cube) either in the absence or in the presence of a “virtual” contact sound, delivered when digits entered the space immediately surrounding the object. Participants were informed in advance whether a contact sound was delivered or not, and the main finding was shorter movement time for the contact sound compared to the no contact sound trials. This result was taken as an evidence of the effect that auditory information might exert on the organization of hand grasping movements. However, the specific reason of why the presence of a contact sound reduced reach duration leaving unchanged hand kinematics was unclear. Furthermore, the delivered contact sound corresponded to the sound of a cork popping out of bottle, a sound which is not normally generated when touching a wooden block. Therefore, the nature of the effect caused by the contact sound remained unexplained.

A subsequent investigation addressed this issue by adopting a similar procedure (Castiello et al., 2010). Here the sound produced by the digits while making contact with objects covered with different materials (i.e., aluminum, paper, wool) was presented before or during the execution of grasping actions performed toward objects covered with the same materials, either in conditions of congruency (e.g., paper sound for grasping a paper object) or incongruency (e.g., paper sound for grasping a wool object). A neutral condition (e.g., a synthetic sound) was also included. The foremost result was that the contact sound delivered either at the beginning or during the movement did affect kinematics. Specifically, both reach duration and the time of hand closure around the visual target decreased when the administered contact sound corresponded to the sound generated by the forthcoming contact with that visual target. Whereas, when the administered contact sound differed from that associated with the interaction between the hand and the visual target, both reach duration and the time of hand closure around the target increased. Therefore, hearing sounds generated during the end part of the action, when the hand touched the visual target-object, had the ability to modulate the “grasp” motor plan selected for that specific target depending on the level of correspondence between the contact sound and the sound produced at touch. Further support to this proposal comes from a second experiment (Castiello et al., 2010). Following a similar procedure, participants were requested to grasp a visual target with the upper and the lower part covered with different materials (e.g., wool and paper, respectively). Also in this case, the task was performed in the presence of a contact sound associated with the material covering one of the two parts of the visual target (e.g., “touching-wool” sound or “touching-paper” sound). Noticeably, when the presented contact sound was “touching- wool” and “touching-paper,” the probability that participants grasped the visual-target object by the wool and the paper surface increased above chance (Figure 1B). How the sound produced by the target object can affect grasping planning has been investigated also by Sedda et al. (2011). In their study, participants had to infer the size of a grasping target relying on the sound produced by placing it within the reaching area, while visual information was varied from trial to trial. The results indicated that participants were able to infer the size of the object, with or without visual information available. The influence of auditory information on action has been demonstrated also in for action observation, showing how the activity of the mirror system can be evoked not only by seeing goal-directed hand actions (di Pellegrino et al., 1992) but also by hearing the sound produced by those actions (Aziz-Zadeh et al., 2004; Fadiga et al., 2005). Activity within the human mirror system has been investigated during the observation of hand actions, while sounds -which could have been either congruent or incongruent with that produced by the observed action- were presented. The results showed enhanced mirror activity in conditions of congruence between visual and auditory stimulation, suggesting that mechanisms similar to those typical for speech perception can arise (Alaerts et al., 2009). Altogether, these findings demonstrate that selection of the “grasp” motor plan to be performed to interact with an object can be influenced by sounds, extending the sensitivity of the mechanism underlying the guidance of actions to the auditory information.

## The Role of Chemosensory Information

**Figure 2:**
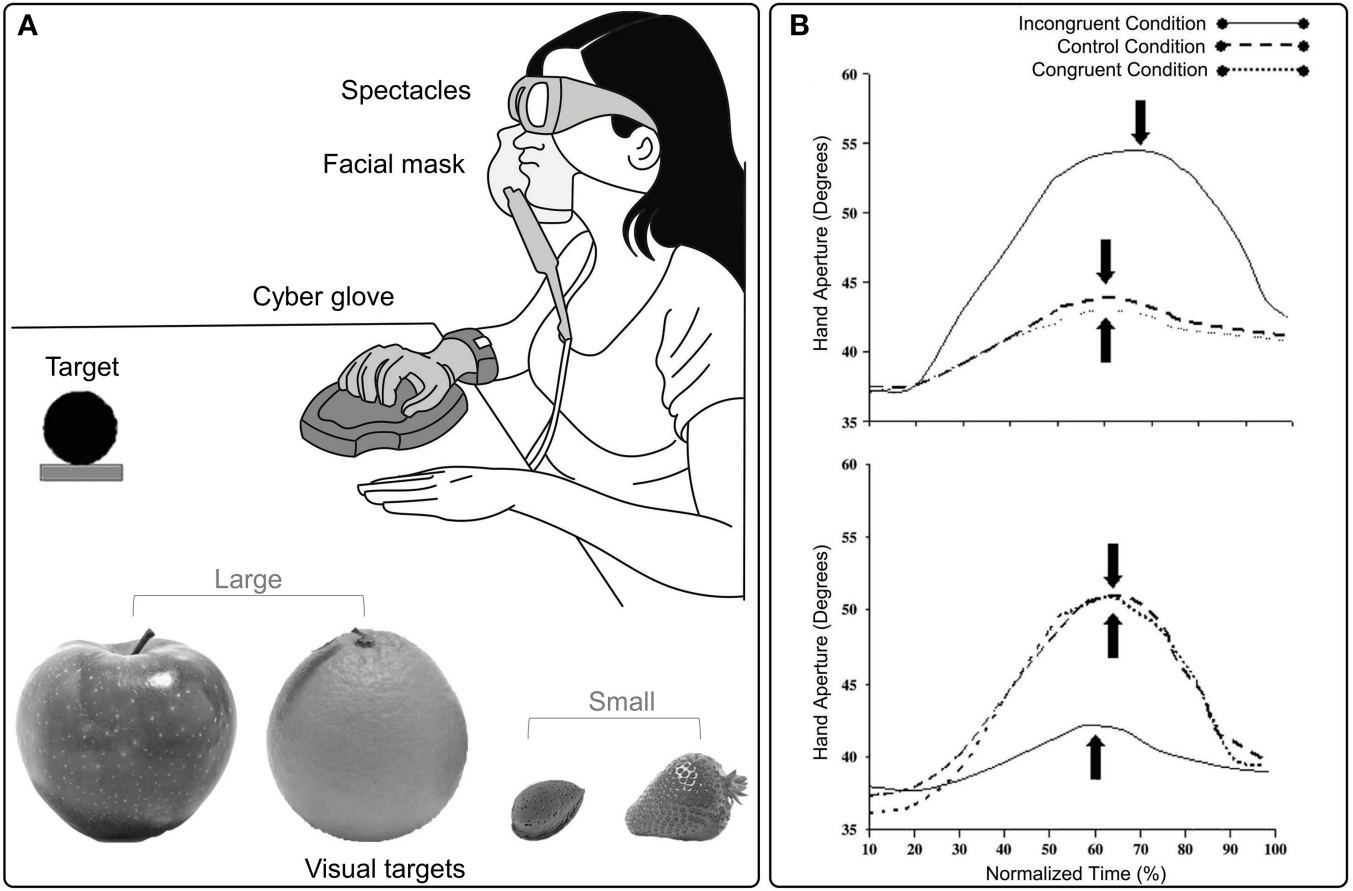
**(A)** The experimental set up and examples of visual targets used in Castiello's et al. (2006) study, in which the existence of cross-modal links between olfaction and vision during grasping movements was investigated. **(B)** Graphical representation of the amplitude and the time (filled arrow) of maximum hand aperture for small (upper panel) and large (lower panel) targets in congruent, incongruent and control odor conditions. The time course of maximum hand aperture is expressed in terms relative to the overall reach duration (%). The amplitude and time of maximum hand aperture is, respectively, greater and delayed for an action toward a small target when olfactory information evokes an object requiring an incongruent large grasp. Conversely, when an action toward a large target is coupled with olfactory information evoking an incongruent small object, maximum hand aperture is smaller and anticipated. Hand aperture reported in the plots is averaged across trials and subjects for each experimental condition.

An aspect which has been largely neglected in terms of the multisensory processes underlying hand grasping movements concerns chemosensory information. To date, only few studies considered reach-to-grasp movements performed toward a visual target-object in the presence of olfactory cues (Castiello et al., 2006; Tubaldi et al., 2008a,b). In these studies, the olfactory stimulus delivered during the grasping action could evoke an object of a smaller or larger dimension than the visual target-object (Figure 2A). This manipulation affected the both the amplitude and the time of maximum hand aperture (i.e., the maximum distance between the thumb and index finger; Figure 2B). In more detail, if the olfactory stimulus evoked an object smaller than the target-object, then the maximum hand aperture was smaller and anticipated in time than when no odor was delivered. If the olfactory stimulus evoked an object larger than the target-object, then the maximum hand aperture was larger and delayed in time than when grasping occurred in the absence of olfactory information (Castiello et al., 2006).

Altogether, the results of these studies indicate that the “size” of an odor influenced the kinematic profile of a reach-to-grasp movement. Crucially, the motor plan evoked by the odor is surprisingly fine grained and when elicited can modulate kinematics patterns both in terms of individual fingers movements and synergic movement amongst digits (Santello and Soechting, 1998). More recently, research on the effect of chemosensory information on reach-to-grasp has been extended to flavor (Parma et al., 2011a,b). In these studies, participants were asked to drink a sip of fruit flavored solution and then reach and grasp a fruit of different size positioned in front of them. The size of the objects (fruit) evoked by the flavor and the size of the visual target could be similar or different in size, and therefore elicit a different kind of grasping. For example, participants could drink a sip of strawberry juice and then reach and grasp an orange: in this case, while the size of the strawberry elicits a precision grip (i.e., the opposition of thumb and index finger), the size of the orange requires a whole-hand grip (i.e., opposition of the thumb to all the other fingers) to be grasped. Overall, the results highlighted how congruence and incongruence between flavor and target size affected kinematic parameters such as the maximum hand aperture. In more detail, a significantly smaller grip aperture was observed when the act of grasping the orange was preceded by a size-incongruent (e.g., strawberry flavored solution) than when it was preceded by a size-congruent (e.g., apple flavored solution) stimulation or water (i.e., control condition). Further, maximum hand aperture was more size attuned when the act of grasping the orange was grasped preceded by a size-congruent stimulation (e.g., orange flavored solution) than when it was grasped preceded by a size-incongruent (e.g., strawberry flavored solution) or water stimulation. The pattern highlighted is in line with studies exploring how interfering perceptual information can modulate grasping components (Castiello, 1999; Patchay et al., 2003, 2006), showing that when considering chemosensory information in combination with visual information, a condition of mismatch can affect the planning and execution of visually guided reach-to-grasp movements (Parma et al., 2011a,b). Rossi et al. (2008) further investigated the role of olfaction in grasping showing that smell can affect the excitability of muscles typically involved in the grasping movement. By means of Transcranial Magnetic Stimulation (TMS) they showed that evoked motor potentials for First Dorsal Interosseous (FDI) and the Abductor Digiti Minimi (ADM) can be modulated by food and non-food odors while participants observed a grasping task. However, this motor facilitation effect was evident only in case of congruence between odor and target of the observed grasping action.

## Conclusion

This review presents the hand as a theoretical vehicle for understanding the multisensory nature of prehensile actions. The hand, an organ through which we explore our social and objective world, is integral to test the multisensory architecture of action. Unveiling how multisensory integration does shape our action not only has important implications for a full understanding of action planning and on-line control, but can also advance knowledge and applications in the fields of motor learning (Sigrist et al., 2013; Luan et al., 2020) and in the development of multisensory wearable systems for rehabilitation of missing or impaired functions (Shull and Damian, 2015). In our daily life we constantly perceive stimuli in their multimodal -rather than unimodal- forms, ending up with multimodal information that shapes and facilitates our actions toward the surrounding world. However, how multiple senses differentially contribute to the formation of a coherent representation of the world and shape our motor behavior is still an under-investigated aspect in motor control research. Nonetheless, the advantage of employing multimodal information overcoming the specific advantage of each modality critically emerges in the development of rehabilitative applications for the replacement or augmentation of impaired functions. Furthermore, deeply comprehending how multisensory integration works in motor control may play a crucial role for the implementation of “tomorrows” hands. It might be surprising that throughout the history of humanoid robot production, attempts to design robots with functional hands have been met with little success. And if the hands are the gateway to the world, it is clear that contemporary research is not yet in a position to provide us with any robots with meaningful active relations. The motor skills of today's best robots are indeed limited in comparison to animals and humans. But, if one were to come about, it would have to behave itself not as a deliberative and precisely calculating machine, but as skillful and dynamic entity in constant adjustment with its environment. A robot of this kind should approximate a reasonable spectrum of different multisensory motor capabilities. The challenge is to determine how multisensory functions can be integrated into meaningful architectures and to test their functional limits. Overall the findings summarized here could also act as a ground for novel motor rehabilitation approaches, exploiting interaction phenomena linking multisensory perception and action in human cognitive and motor system.

## Author Contributions

SB, UC, and CB identified the topic, performed the literature overview, and wrote the manuscript. All authors contributed to the article and approved the submitted version.

## Conflict of Interest

The authors declare that the research was conducted in the absence of any commercial or financial relationships that could be construed as a potential conflict of interest.
